# Aberrant Expressional Profiling of Known MicroRNAs in the Liver of Silver Carp (*Hypophthalmichthys molitrix*) Following Microcystin-LR Exposure Based on samllRNA Sequencing

**DOI:** 10.3390/toxins12010041

**Published:** 2020-01-09

**Authors:** Yiyi Feng, Xi Chen, Junguo Ma, Bangjun Zhang, Xiaoyu Li

**Affiliations:** College of Life Science, Henan Normal University, Xinxiang 453007, China; fengyiyi101825@126.com (Y.F.); xxchen1108@163.com (X.C.); mjunguo_1378@126.com (J.M.); zhangbangjun@htu.edu.cn (B.Z.)

**Keywords:** Microcystin-LR, miRNA, Illumina sequencing, silver carp, hepatotoxicity

## Abstract

Microcystin-LR (MC-LR) poses a serious threat to human health due to its hepatotoxicity. However, the specific molecular mechanism of miRNAs in MC-LR-induced liver injury has not been determined. The aim of the present study was to determine whether miRNAs are regulated in MC-LR-induced liver toxicity by using high-throughput sequencing. Our research demonstrated that 53 miRNAs and 319 miRNAs were significantly changed after 24 h of treatment with MC-LR (50 and 200 μg/kg, respectively) compared with the control group. GO enrichment analysis revealed that these target genes were related to cellular, metabolic, and single-organism processes. Furthermore, KEGG pathway analysis demonstrated that the target genes of differentially expressed miRNAs in fish liver were primarily involved in the insulin signaling pathway, PPAR signaling pathway, Wnt signaling pathway, and transcriptional misregulation in cancer. Moreover, we hypothesized that 4 miRNAs (miR-16, miR-181a-3p, miR-451, and miR-223) might also participate in MC-LR-induced toxicity in multiple organs of the fish and play regulatory roles according to the qPCR analysis results. Taken together, our results may help to elucidate the biological function of miRNAs in MC-LR-induced toxicity.

## 1. Introduction

Cyanobacteria are highly adaptable and widely distributed photosynthetic bacteria in almost all environments [1]. In the past several decades, global warming and overinvestment in nutrients have both led to the frequency and intensity of large algal blooms [2]. Cyanobacterial blooms in freshwater systems can produce multiply harmful cyanotoxins, particularly microcystins (MCs), which are mainly produced by bloom-forming *Microcystis aeruginosa* in freshwater. MCs are a group of potent toxins that become a threat to public health when cell-bound MCs are significantly released from the dying *Microcystis* into the water column [3]. MCs have at least 246 variants according to their chemical structure, a heptapeptide ring [4]. The most abundant, best known, and most studied congener MC form is microcystin-LR (MC-LR), which has leucine (L) and arginine (R) as variable amino acid residues [4,5]. The unique cyclic structure of MCs makes them less vulnerable to physical and chemical breakdown in natural environments, including sunlight, extreme pH, and high temperatures [6,7]. The frequent occurrence and accumulation of microcystins in drinking water poses a serious threat to human health [8]. After entering the human body, MCs bind to organic anion transporter polypeptides (OATPs), which inhibit the activities of protein phosphatases 1 and 2a, leading to phosphorylation disorders of their substrates [9]. Around the world, many large freshwater lakes and rivers are experiencing increasingly severe cyanobacterial blooms, such as Lake Erie and Monterey Bay in the USA [10,11], the southern Adriatic Sea in Italy [12], Lake Suwa in Japan [13], Tabocas Reservoir in Brazil [14], Lake Alexandrina in Australia [15], and Lakes Poyang [16], Chaohu [17], Dianchi [18], and Erhai [19] in China. MC-LR has been shown to be a tumor promoter that is fatal to animals and has been associated with the development of primary liver cancer in humans. Moreover, epidemiological investigations have found that chronic exposure to *Microcystis* is associated with increased rates of liver and colorectal cancer through consumption of contaminated drinking water and aquatic foods [20,21].

A class of naturally occurring small noncoding RNAs with lengths of 18–24 nucleotides (18–24 nt) are called microRNAs (miRNAs), which are produced by subsequent processing steps mediated by members of the RNase III family, Dicer, and Drosha proteins [22,23]. Cumulative studies have shown that miRNAs can control gene expression post-transcriptionally by binding to the 3’ UTR sequences of mRNAs and are implicated in many physiological processes, including apoptosis, immunoprotection, nervous system development, and cancer pathogenesis [24,25,26]. Recently, changes in miRNA expression in various cancers have been reported, and tissue miRNA profiles show strong potential for application in cancer definitions [27,28]. Recently, there have been increasing numbers of studies investigating the function of miRNA in MC-LR toxicity. The results of Xu et al. suggested that chronic MC-LR exposure alters the miRNA expression profile of WRL-68 cells and causes phenotypic transformation [29]. A report showed that miR-451a may protect against MC-LR-induced DNA damage by downregulating the expression of p-AKT1 [30]. In addition, previous studies have shown that the computational analysis of small RNA sequencing datasets is a powerful tool to identify miRNAs associated with MC-LR toxicity. For example, Li et al. identified MC-LR-induced female mammals’ reproductive toxicity-related microRNAs in mouse granulosa cells using bioinformatics analysis [31]. Qu et al. identified liver detoxification-related microRNAs to investigate the antitoxic effects of MC-LR in fish by next-generation sequencing [32]. Therefore, exploring the functions and expression levels of miRNAs could provide valuable and novel insights into MC-LR toxicology. Meanwhile, this study provides new theoretical support for discovering new molecular markers for detecting liver toxicity of MCs and new drug targets in the treatment of hepatitis and liver cancer.

## 2. Results

### 2.1. Solexa Sequencing of Small RNAs

Through Solexa high-throughput sequencing, 11,452,259 total reads, 12,024,799 total reads, and 11,637,795 total reads from three libraries were obtained. The sequence length distributions of the three libraries were not significantly different; most of the sequences (87.13%, 89.80%, and 87.24%) were between 21 and 23 nucleotides (Supplementary Materials Figure S1). The length of the cleaned reads peaked at 22 nt (Supplementary Materials Figure S1). After removing the 5’ and 3’ adapters, contaminate reads, and reads less than 18 nucleotides, 11,152,117 high-quality, 11,799,758 high-quality, and 11,442,649 high-quality clean reads for three groups were extracted and annotated with NCBI GenBank and the RFam 10.1 database (Supplementary Materials Table S1-1). Approximately 8.45%, 10.85%, and 10.9% of sRNA could be mapped to the genome in the three groups via SOAP or bowtie software (Supplementary Materials Table S1-2). Reads of rRNA, tRNA, snoRNA, and other snRNAs were annotated and then removed from the following analysis (Supplementary Materials Figure S2). The sRNA and miRBase databases were aligned by blast or bowtie to identify known miRNAs for subsequent analysis (Supplementary Materials Table S1-3).

### 2.2. Differentially Expressed Known miRNAs

To explore the toxic effects of low-dose and high-dose MC-LR on sequencing, we selected concentrations of 50 and 200 μg/kg in subsequent microRNA analyses. As shown in Supplementary Materials Figure S3, 53 miRNAs and 319 miRNAs were significantly changed after 24 h of exposure to MC-LR (50 and 200 μg/kg, respectively) compared with the control group (Supplementary Materials Figure S3). Among the 53 miRNAs, 38 were upregulated, while 15 were downregulated. Among 319 miRNAs, the expression of 227 was upregulated, while the expression of 29 was downregulated. Compared with the MC-50 group, 203 miRNAs were significantly upregulated, and 163 miRNAs were downregulated in the MC-200 group (Supplementary Materials Table S2).

### 2.3. GO Analysis of the Candidate Target Genes of Differentially Expressed miRNAs

**Figure 1:**
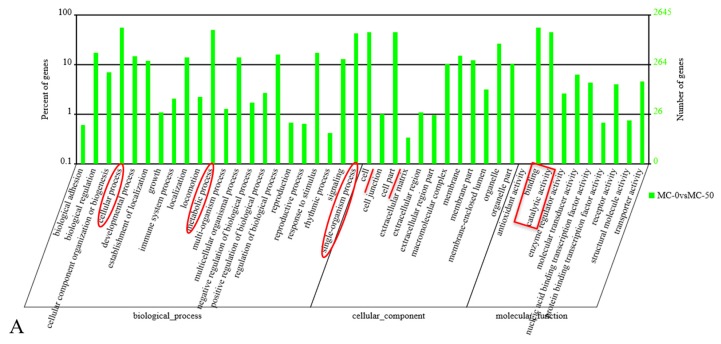

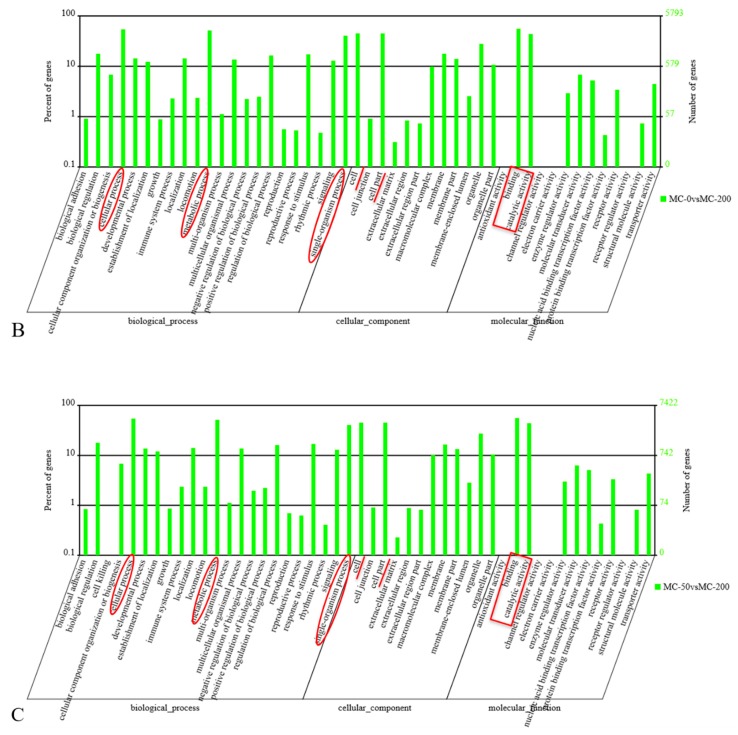
GO function classification target genes of known miRNAs in the liver of silver carp after exposure to microcystin-LR (MC-LR). Abscissa: class classification. The three different classifications represent the three basic classifications of GO terms. From left to right they are biological process, cellular component, and molecular function. Left ordinate: the ratio of the number of candidate target genes annotated to the term (including the subterms of the term) to the total number of candidate target genes annotated. Right ordinate: the number of candidate target genes annotated to the term (including the subterm of the term). (**A**) MC-0 vs. MC-50; (**B**) MC-0 vs. MC-200; (**C**) MC-50 vs. MC-200.

The distribution of candidate target genes in the gene ontology was compared with the reference group, the number of genes of the significantly enriched GO term (functional items) was counted (as shown in Figure 1), and candidate target genes were screened to determine which biological functions were significantly correlated and to identify the main biological functions performed by candidate target genes. GO enrichment analysis showed that these target genes were related to cell, cell part, binding, catalytic activity, cellular process, metabolic process and single-organism process (Figure 1A, Supplementary Materials Table S3 MC-0 vs. MC-50, Figure 1B, Supplementary Materials Table S3 MC-0 vs. MC-200, Figure 1C, Supplementary Materials Table S3 MC-0 vs. MC-200).

### 2.4. KEGG Pathway Analysis of the Candidate Target Genes of Differentially Expressed miRNAs

**Figure 2:**
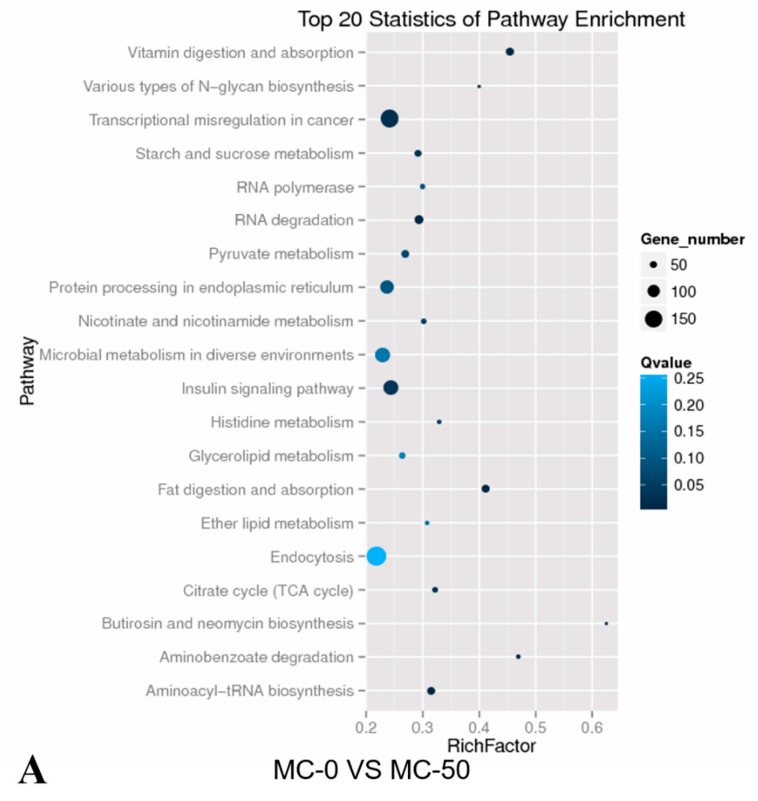

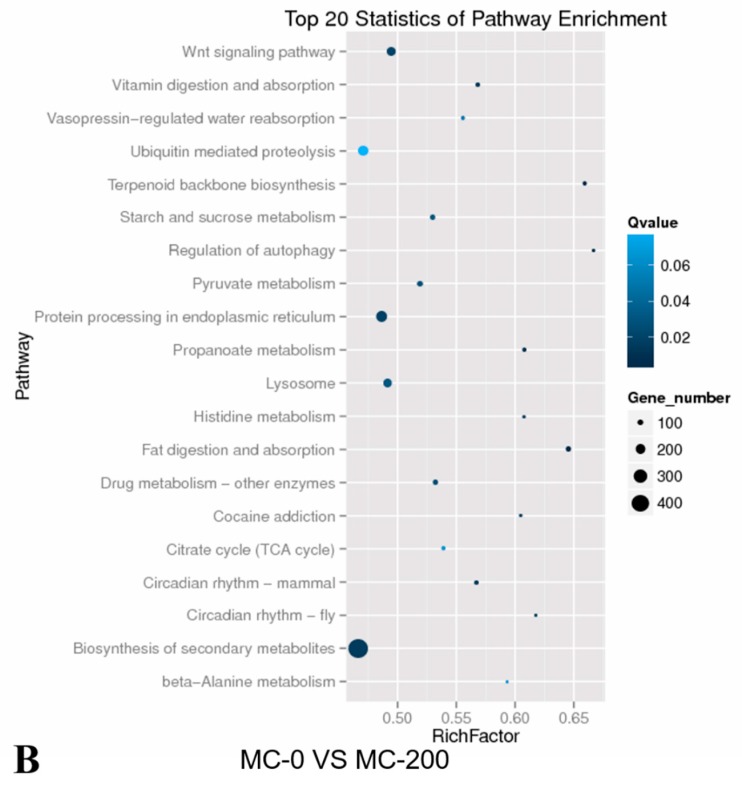

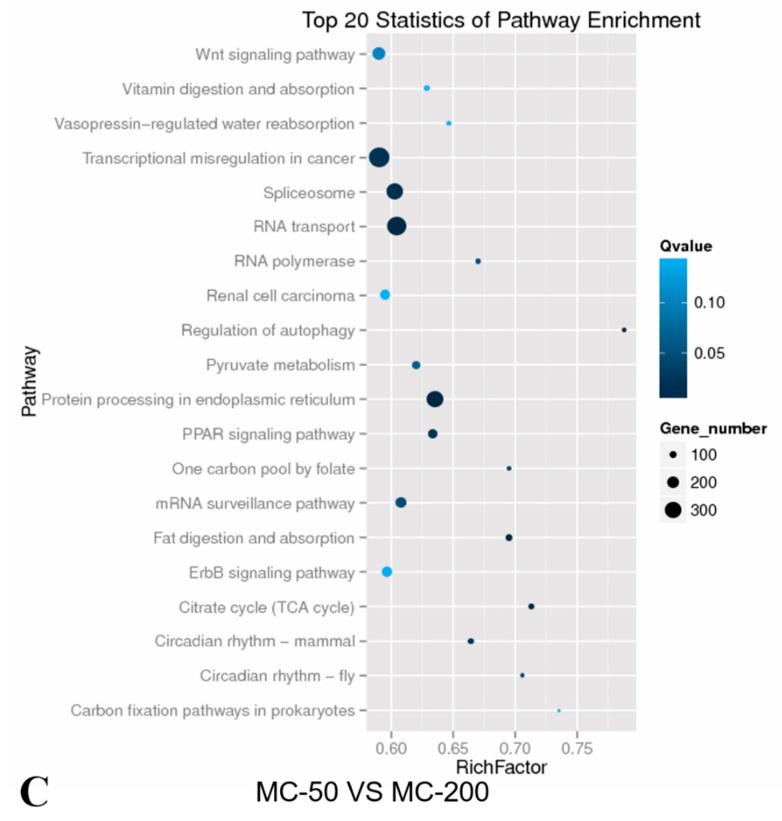
KEGG pathway analysis of the target genes of known miRNAs in the liver of silver carp after exposure to MC-LR. Horizontal axis: Rich factor. The larger the point, the higher the enrichment degree, the more candidate target genes in this pathway, and the color of the point corresponds to a different q value range. Vertical axis: The name of the pathway. (**A**) MC-0 vs. MC-50; (**B**) MC-0 vs. MC-200; (**C**) MC-50 vs. MC-200.

To identify biological pathways in which differentially expressed miRNAs were involved in the hepatotoxicity of MC-LR induced in silver carp, the target genes were mapped to the reference pathways recorded in the KEGG database. The KEGG pathway analysis revealed 20 major pathways occupied by the most abundant target gene counts of differentially expressed miRNAs. In the MC-0 vs. MC-50 group, the target gene enrichment pathways included transcriptional misregulation in cancer, the insulin signaling pathway, protein processing in the endoplasmic reticulum, microbial metabolism in diverse environments, and endocytosis (Figure 2A). In group MC-0 vs. MC-200, the target gene enrichment pathways included biosynthesis of secondary metabolites, protein processing in the endoplasmic reticulum, the Wnt signaling pathway, drug metabolism—other enzymes, pyruvate metabolism, lysosomes, and ubiquitin-mediated proteolysis (Figure 2B). In the MC-50 vs. MC-200 group, the target gene enrichment pathways included protein processing in the endoplasmic reticulum, RNA transport, spliceosomes, transcriptional misregulation in cancer, the PPAR signaling pathway, the mRNA surveillance pathway, pyruvate metabolism, the Wnt signaling pathway, the ErbB signaling pathway, and renal cell carcinoma (Figure 2C). All these results revealed the potential function of miRNA targets, which may form a regulatory network and execute a vital role in fish physiology (Supplementary Materials Table S4).

### 2.5. Confirmatory Study and Additional Profiling of the Selected miRNAs by qPCR

**Figure 3:**
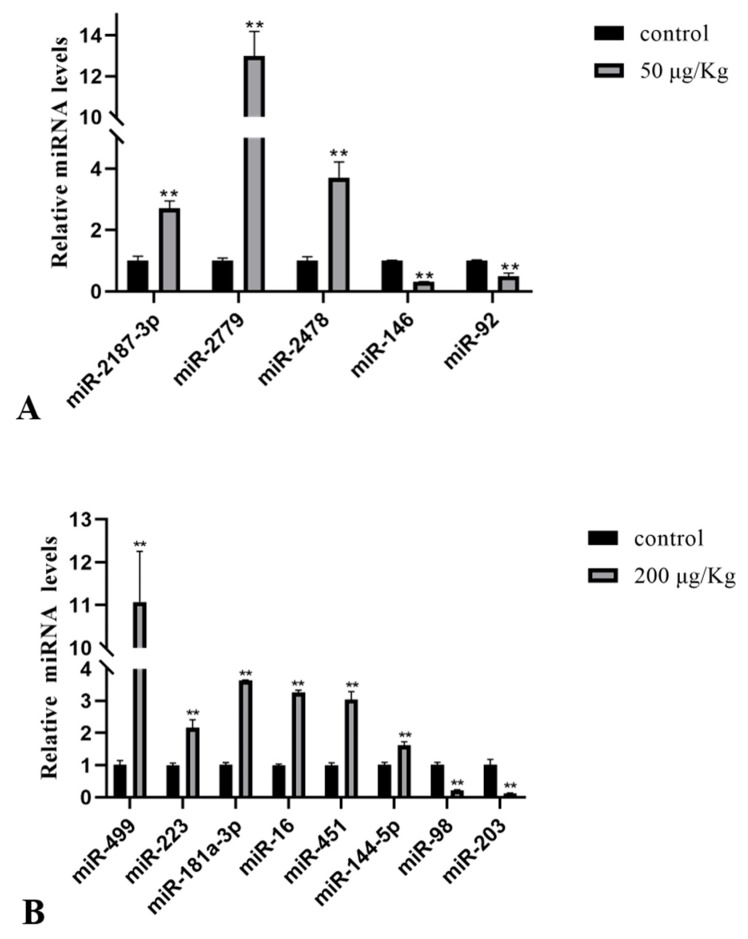
Validation of the known differentially expressed miRNAs. The asterisk indicates a significantly different response from the control group (** *p* < 0.01). (**A**) Relative levels of differentially expressed miRNA after 50 μg/kg MC-LR exposure; (**B**) Relative levels of differentially expressed miRNA after 200 μg/kg MC-LR exposure.

Some of the differentially expressed miRNAs in the liver were verified using qPCR, and we found that the verification results were consistent with the sequencing results. As depicted in Figure 3, qPCR analysis of 50 μg/kg MC-LR showed that the expression of 3 miRNAs (miR-2187-3p, miR-2779, and miR-2478) was upregulated, while the expression of 2 miRNAs (miR-146 and miR-92) was downregulated (Figure 3A). Data from the 200 μg/kg MC-LR treatment groups showed that the expression levels of miR-16, miR-144-5p, miR-181a-3p, miR-223, miR-451, and miR-499 were upregulated, while the expression levels of miR-203 and miR-98 were downregulated (Figure 3B).

### 2.6. Expression of Selected miRNAs in the Liver of Silver Carp

**Figure 4:**
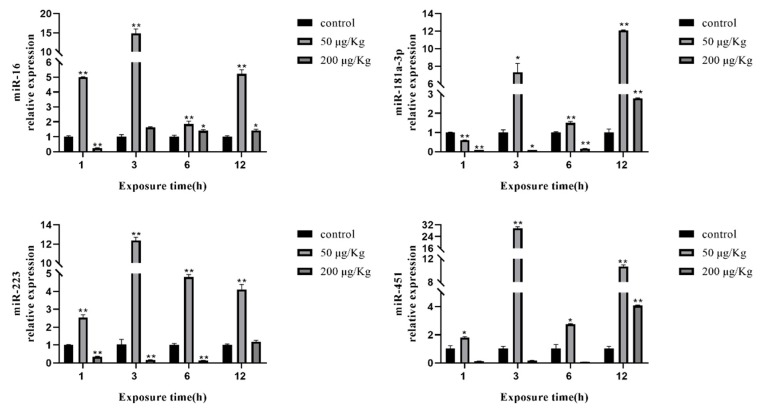
Expression of miRNAs in the liver of silver carp after exposure to MC-LR for 1, 3, 6, and 12 h. The asterisk indicates a significantly different response from the control group (* *p* < 0.05, ** *p* < 0.01).

In addition, we examined the expression of miR-16, miR-181a-3p, miR-223, and miR-451 in the liver of silver carp during the early stages of MC-LR exposure. After MC-LR exposure for 1, 3, 6, and 12 h, the expression of four miRNAs was significantly upregulated in the low-concentration group, except that the expression level of miR-181a-3p was downregulated at 1 h after MC-LR exposure. However, the expression levels of miR-181a-3p, miR-223, and miR-451 were significantly decreased in the high-concentration group after MC-LR exposure for 1, 3, and 6 h, except for miR-16. With the extension of exposure time, the expression of four miRNAs was upregulated at 12 h of exposure to MC-LR (Figure 4). 

### 2.7. Expression of Selected miRNAs in Different Tissues of Silver Carp after Exposure to MC-LR

**Figure 5:**
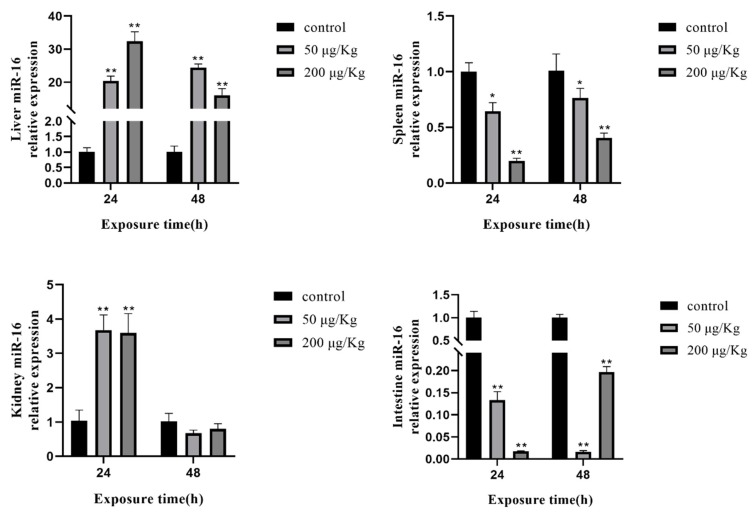
Expression of miR-16 in different tissues of silver carp after MC-LR exposure. The asterisk indicates a significantly different response from the control group (* *p* < 0.05, ** *p* < 0.01).

Moreover, we detected the expression of four selected miRNAs in different tissues of silver carp after MC-LR exposure. The expression of miR-16 was clearly upregulated in the liver of silver carp. Conversely, the expression levels of miR-16 in the spleen and intestine were significantly downregulated. However, the change in miR-16 was increased in the kidney at 24 h (Figure 5).

**Figure 6:**
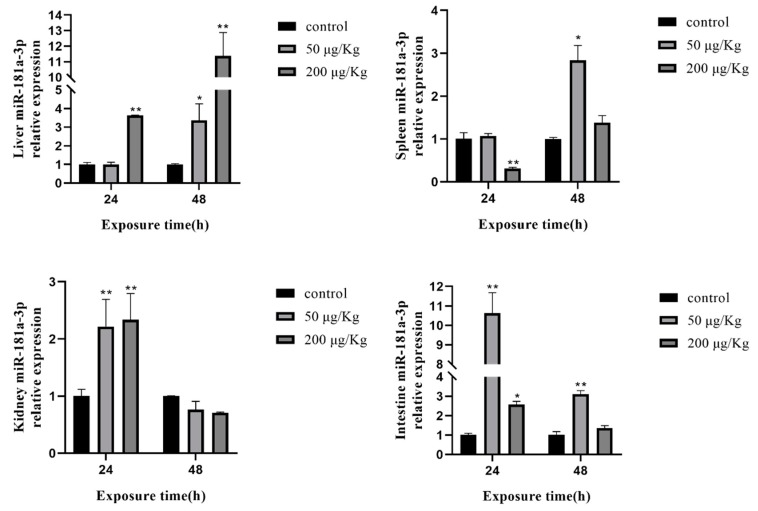
Expression of miR-181a-3p in different tissues of silver carp after MC-LR exposure. The asterisk indicates a significantly different response from the control group (* *p* < 0.05, ** *p* < 0.01).

In the liver, the expression of miR-181a-3p was upregulated at 24 and 48 h, except for the low-concentration group at 24 h. In the spleen, the change in miR-181a-3p was on the rise. Although the expression of miR-181a-3p was downregulated at 24 h in the high-concentration group, the expression levels of miR-181a-3p had increased at 48 h. In the kidney, miR-181a-3p changed in line with miR-16. In contrast, the expression of miR-181a-3p was upregulated in the intestine (Figure 6). 

**Figure 7:**
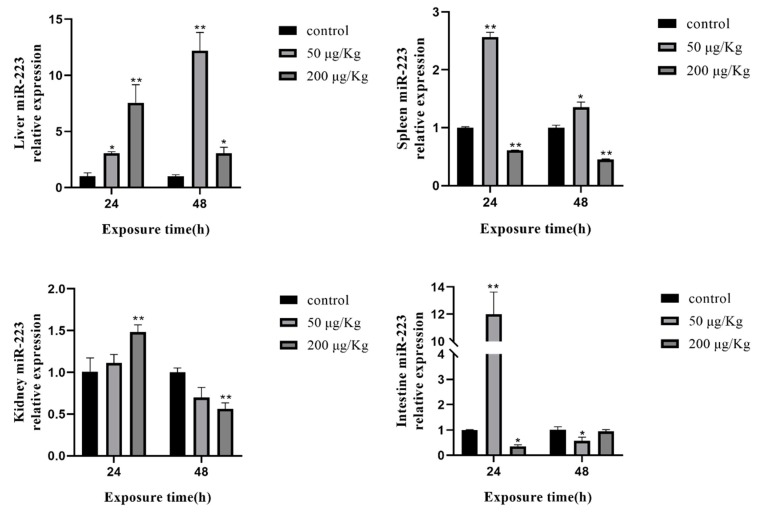
Expression of miR-223 in different tissues of silver carp after MC-LR exposure. The asterisk indicates a significantly different response from the control group (*p < 0.05, ** *p* < 0.01).

The expression of miR-223 was clearly increased in the liver, as well as miR-16. In the spleen, the expression of miR-223 was upregulated in the low-concentration group and downregulated in the high-concentration group. In the kidney, miR-223 changed only in the high-concentration group. Compared with the control group, the expression levels of miR-223 were increased by approximately 12 times in the intestine at 24 h after treatment with the low concentration of MC-LR (Figure 7). 

**Figure 8:**
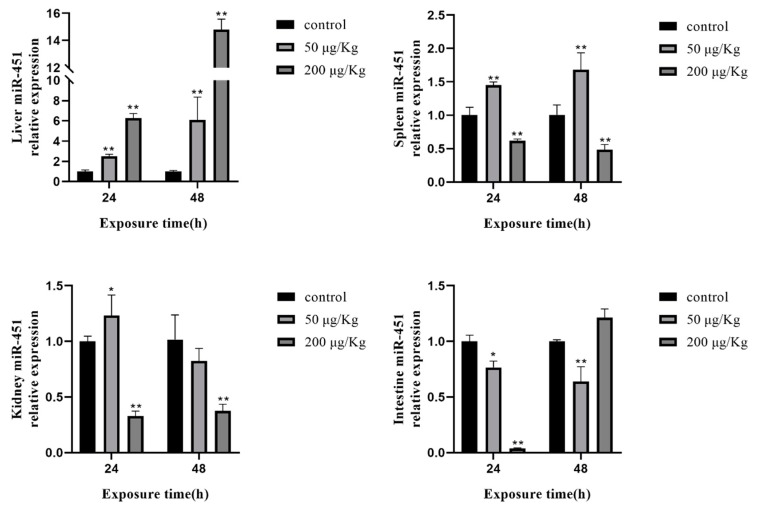
Expression of miR-451 in different tissues of silver carp after MC-LR exposure. The asterisk indicates a significantly different response from the control group (* *p* < 0.05, ** *p* < 0.01).

The expression of miR-451 was clearly increased in the liver, as well as miR-16 and miR-223. The expression of miR-451 showed the same change as miR-223 in the spleen. In the kidney, the expression of miR-451 was increased at 24 h in the low-concentration group and decreased at 24 and 48 h in the high-concentration group. In the intestine, the change in miR-451 was significantly downregulated, except that there was no significant change at 48 h in the high-concentration group (Figure 8).

## 3. Discussion

MCs are known to be widespread in freshwater where cyanobacteria bloom, and they are highly toxic to aquatic organisms and even humans [33]. Biological and pathological processes are assumed to be due to exposure to MC-LR and associated changes in miRNA expression levels, including metabolism, developmental timing, signal transduction, cell proliferation, differentiation, apoptosis, cancer progression, and tumorigenesis [34,35]. Meanwhile, miRNAs have been proven to regulate multiple molecular functions and have been correlated with various diseases [36,37]. The multiple roles of miRNAs have generated interest in using miRNAs as potential diagnostic and prognostic biomarkers to detect MC-LR toxicity and liver damage in fish [38,39,40]. Hence, we performed high-throughput sequencing to detect the expression of miRNAs in silver carp at 24 h after MC-LR exposure to identify which miRNAs play a key regulatory role in MC-LR toxicity.

The results revealed that the expression of 53 miRNAs and 319 miRNAs significantly changed after 24 h of exposure to MC-LR (50 μg/kg and 200 μg/kg) compared with the control group. Compared with the MC-50 group, the expression of 203 miRNAs was significantly upregulated, and 163 miRNAs were downregulated in the MC-200 group. Moreover, we found that MC-LR exposure accelerated the expression of miR-2187-3p, miR-2779, miR-2478, miR-16, miR-144-5p, miR-181a-3p, miR-223, miR-451, and miR-499, while miR-146, miR-92, miR-203, and miR-98 were suppressed when compared to the control group. The qPCR results confirmed the above results, suggesting that these miRNAs may be involved in the hepatotoxicity of MC-LR. Ma et al. confirmed that 21 and 37 miRNAs were altered in HepG2 cells after MC-LR exposure (10 and 50 µM) [35]. Yang reported that treatment of the HL7702 cell line with MC-LR (1, 2.5, 5, or 10 µM) clearly altered the expression of 3, 10, 9, and 99 miRNAs, respectively, when compared with control cells [34]. Therefore, it is reasonable to believe that miRNAs play a regulatory role after MC-LR exposure.

The identification and analysis of differentially expressed miRNA target genes is a key step to better understand the molecular functions of miRNAs [41]. In this study, GO enrichment analysis and KEGG pathway analysis were performed on these predicted target genes to determine the active regulation functions and pathways of miRNA in silver carp liver after MC-LR exposure. GO enrichment analysis showed that these target genes were associated with metabolic processes, cellular processes, and single-organism processes. Furthermore, KEGG pathway analysis demonstrated that the target genes of differentially expressed miRNAs in the liver predominantly participated in the insulin signaling pathway, PPAR signaling pathway, mRNA surveillance pathway, Wnt signaling pathway, and transcriptional dysregulation in cancer. Understanding abnormal insulin signaling is an important goal because it can lead to a range of neurodegeneration, female infertility, kidney disease, blindness, stroke, cardiovascular disease, hypertension, and systemic disorders—dyslipidemia [42]. The PPAR signaling pathway regulates the maintenance of metabolic homeostasis and inflammatory gene expression, lipid metabolism, and lipogenesis and induces anticancer activity in a diversity of cancers. PPAR transcriptional activity is regulated by nongene cross position with phosphatases and kinases, including AMPK, PKC, PKA, p38-MAPK, GSK3, and ERK1/2 [43]. The Wnt signaling pathway widely exists in invertebrates and vertebrates and is a highly conserved signaling pathway in the evolution of species. Wnt signaling plays an important role in the early development of organ formation, tissue regeneration, animal embryos, and other physiological processes. If mutations in key proteins in this signaling pathway lead to abnormal activation, cancer may be induced [44,45]. In summary, target genes of differentially expressed miRNAs induced by MC-LR may be involved in alterations in insulin signaling, PPAR, and Wnt pathways, indicating that MC-LR-induced toxicity in the liver of silver carp might be related to these pathways.

Quantifying miRNAs in different stages of MC-LR exposure is an important initial step to explore the functions of miRNAs. According to the sequencing results, we found that the mature sequences of four miRNAs (miR-16, miR-181a-3p, miR-223, miR-451) were conserved in different species and had different expression levels. Subsequently, we examined the abundance of four selected differentially expressed miRNAs (miR-16, miR-181a-3p, miR-223, miR-451) by real-time qPCR in the liver of silver carp exposed for 1, 3, 6, and 12 h to MC-LR (50 and 200 µg/kg); miR-16 was found to act as a tumor suppressor associated with the development of chronic lymphocytic leukemia, breast cancer, lung cancer, and colorectal cancer [46,47]. Accumulated evidence has shown that the expression of miR-16 is decreased in multiple cancer cells, and elevated expression of miR-16 significantly inhibits the proliferation of cancer cells and induces G0/G1 cell cycle arrest [48]. miR-181a plays a key role in the immune response by regulating T cells [49,50]. In addition, studies have suggested that miR-16-5p might be a critical factor involved in the anti-inflammatory effects [51,52]. In the present study, the expression of miR-16 and miR-181a-3p was clearly increased in the early stages of MC-LR exposure. The results suggested that miR-16 and miR-181a-3p may participate in the MC-LR-induced inflammatory response. Wei et al. found that miR-223 acts as a potential tumor marker and inhibits FOXO1 in breast cancer [53]. Pulikkan et al. reported that ectopic miR-223 expression suppressed tumorigenesis by regulating the G1/S cell cycle phase transition by degrading E2F1 [54]. Moreover, miR-223, a commonly repressed miRNA in hepatocellular carcinoma cells, has been confirmed to be involved in many important physiological and pathological processes, including stemness maintenance, metastasis, and proliferation in HCC [55,56]. Numerous studies have confirmed that miR-451 is involved in biological mechanisms, including differentiation and development, cell cycle and proliferation, cell survival, and apoptosis [57]. There is evidence for roles of miR-451 in the regulation of multiple signaling pathways, including the Wnt signaling, AMPK signaling, and IL-6R-STAT3 pathways [58,59,60]. Hence, there is reason to believe that miR-223 and miR-451 can be promising cancer biomarkers with therapeutic potential. To sum up, we have reason to believe that miR-223 may be involved in the toxic effects of MC-LR by regulating cell cycle changes, while miR-451 may be involved in MC-LR toxicity by regulating multiple signaling pathways.

Tissue-specific expression of miRNAs provides an essential reference to analyze variation of miRNA expression under various physiological conditions [61]. Therefore, the expression levels of these four miRNAs were detected in different tissues (liver, spleen, kidney, and intestine) of silver carp after MC-LR exposure. The expression levels of miR-16, miR-181a-3p, miR-223, and miR-451 were promoted in the liver of silver carp. However, the changes in these miRNAs were diverse in the spleen, kidney, and intestine of silver carp after MC-LR exposure. Earlier studies confirmed the nephrotoxicity and hepatotoxicity of MCs [62,63]. Furthermore, accumulating evidence has indicated that the gonads might be another important target organ of MC toxicity [64,65,66]. In conclusion, we speculated that these four miRNAs may be involved in MC-LR-induced toxicity in multiple organs.

## 4. Conclusions

The present study reveals the comprehensive miRNA expression profiles of silver carp liver and provides biological knowledge for the investigation of miRNA-dependent pathways in fish following MC-LR exposure. These results suggest that miRNA might play an important negatively regulated role in MC-LR toxicity. Moreover, we speculate that four miRNAs (miR-16, miR-181a-3p, miR-223, miR-451) may also be involved in MC-LR toxicity in multiple organs.

## 5. Materials and Methods

### 5.1. Ethics Statement

All fish were handled strictly according to the requirements of guide to ethical review of the welfare of laboratory animals of the People’s Republic of China (Draft of Laboratory Animal Welfare and Ethics Committee in China released on February 6, 2018; identification code: GB/T 35892—2018; date of approval: 1 September 2018).

### 5.2. Fish and Treatment

Juvenile silver carp (*Hypophthalmichthys molitrix*) (mean body length, 16 ± 0.5 cm; mean body weight, 37.5 ± 3 g) used in this study were purchased, cultured, and treated as described previously [67].

Silver carp (in total 6 per condition) were exposed to 0 (control), 50 μg/kg (low concentration), or 200 μg/kg (high concentration) in separate flow-through tanks. Standard MC-LR (C_49_H_74_N_10_O_12_, ≥95% purity, HPLC) was purchased from Express Technology Co., Ltd., Beijing, China.

For the exposure, all fish received a single intraperitoneal injection of the MC-LR solution (treatment) or the same volume of saline solution alone (control). The fish from two experimental groups and one control group were kept in separate flow-through tanks. When sampling, six healthy individuals were randomly taken from each of the experimental groups after 1, 3, 6, 12, 24, and 48 h of exposure.

### 5.3. Tissue Collection and RNA Isolation

After exposure, various fish tissues (liver, spleen, kidney, and intestine) were separately collected from each treatment group. Small RNA was isolated using TRIzol reagent (Cwbiotech, Beijing, China) following the instructions of the manufacturer. The Agilent 2100 bioanalyzer (Agilent, Germany) was used to detect the quality of small RNA. If the quality of small RNA met the requirements, transcriptome analysis could be performed by small RNA sequencing.

### 5.4. Small RNA Library Construction and Sequencing

Three small RNA libraries (MC-0, MC-50, MC-200) were constructed for liver from silver carp in each treatment group (0, 50 μg/kg, 200 μg/kg MC-LR). After RNA quality control, the samples were sequenced at the Beijing Genomics Institute (BGI), Shenzhen, China, using Illumina HiSeq technology. The smallRNA (sRNA) obtained by HiSeq deep sequencing covers almost all RNA, including miRNA, siRNA, piRNA, rRNA, tRNA, snRNA, snoRNA, repeat associate sRNA, and exon or intron degradation fragments. Further analysis was performed using clean sequence reads obtained from Solexa sequencing.

### 5.5. Sequence Data Analysis

The 49-nt sequence obtained by HiSeq sequencing was obtained by filtering to obtain a reliable target sequence, and the quality, length, and common sequence between the samples were counted. The sRNA was mapped to the genome by SOAP or bowtie to analyze the expression and distribution of sRNA in the genome. The sRNA and miRbase databases were aligned by blast or bowtie to identify known miRNAs that can be used for subsequent analysis. The sRNA was subjected to a repeat annotation by the program to screen and remove the sequences associated with the repeat. The sRNA and GenBank databases and the sRNA and Rfam databases were aligned by blast or bowtie to screen and remove sequences associated with rRNA, scRNA, snoRNA, snRNA, tRNA, etc. The sRNAs were aligned to exons and introns to screen and remove sequences associated with exons and introns. By sorting the annotation of the target sequence, information on each component and expression amount contained in the sample could be obtained. In each of the above annotation information, it was possible that there was a case where one sRNA was simultaneously compared with a plurality of different annotation information. To have a unique annotation for each unique sRNA, the sRNA was traversed according to the priority order of rRNA > known miRNA > piRNA > repeat > exon > intron, and sRNA with no annotation information was represented by unann. Based on the identification of miRNAs, a flexible differential analysis strategy for actual sample information could be used to find miRNAs with different expressions between different groups or between different individuals. 

### 5.6. Differential miRNA Expression Analysis

miRNA expression was normalized in the three libraries (MC-0, MC-50, and MC-200) to obtain transcript expression per million using the following formula: normalized expression = (number of actual microRNA/total cleaning reads) × 1,000,000 [68]. To screen for differentially expressed miRNAs, we compared the miRNA expression of the three libraries.

### 5.7. miRNA Verification and Analysis by Quantitative Real-Time PCR

Quantitative real-time PCR (qPCR) was performed to profile the expression levels of miRNAs according to the manufacturer’s instructions (Cwbiotech, Beijing, China). The miRNA-specific primers were designed with primer software based on identified miRNA sequences. Relative expression levels of the miRNA were measured in terms of threshold cycle value (Ct) and were normalized to U6 snRNA using the equation 2^−ΔΔCt^. All primers were synthesized by GENEWIZ, China, and are shown in Table 1. 

### 5.8. Known miRNA Target Gene Prediction and Function Analysis

Targets of the differentially expressed miRNAs were predicted by miRanda and TargetScan software. TargetScan first searches for the perfect complement of the seeds and then calculates the context score based on the type of surrounding sites [69]. MiRanda improved the accuracy of predictions by measuring the complementary fraction of miRNA binding sites [70]. KEGG is a major public database related to pathways that systematically analyzes the metabolic pathways of gene products in cells, as well as the functions and annotations of these gene products. In organisms, different genes cooperate with each other to exert their biological functions. Through pathway significant enrichment analysis, the most important biochemical metabolic pathways and signal transduction pathways that candidate target genes participate in can be determined, which helps to further understand the biological functions of genes. To further understand the biological functions of the identified miRNAs, we annotated miRNAs and their target genes through GO analysis and KEGG pathway analysis.

### 5.9. Statistical Analysis

All values were processed as the mean ± standard deviation. Statistical analyses of the data were analyzed using SPASS 20.0 Significant differences between the MC-LR challenged groups and the control group were compared by one-way analysis of variance (ANOVA). Significant differences are denoted by * *p* < 0.05, and extremely significant differences are indicated by ** *p* < 0.01.

## Figures and Tables

**Table 1:** Sequences of miRNA primers.

Gene	Forward Primer (5′–3′)	Reverse Primer (5′–3′)
U6	GCTTCGGCAGCACATATACTAA	GCTTCACGAATTTGCGTGTCAT
miR-2779	ATCCGGCTCGAAGGACTT	
miR-2478	GGTCCCACTTCTGACACCAT	
miR-2187-3p	GGGCAGGCTATGCTAATCTATG	
miR-146	GGGTGAGAACTGAATTCCATAG	
miR-92	ATATTGCACTCGTCCCGGC	
miR-499	GACTTGCAGTGATGTTTAGAG	
miR-203	GTTTAGGACCACTTGATCAGGG	
miR-451	GGGCCGTTACCATTACTGAGT	
miR-181a-3p	ACCATCGACCGTTGATTGTACC	
miR-223	GGTCAGTTTGTCAAATACCCCA	
miR-144-5p	GGGACAGGATATCATCGTATACTG	
miR-16 miR-98	GGCTAGCAGCACGTAAATATTGG GTTGGGGTGAGGTAGTAAGTTGT	

## Supplementary Materials

The following are available online at https://www.mdpi.com/2072-6651/12/1/41/s1, Figure S1: Small RNA length distribution. Abscissa: fragment length. Ordinate: proportion of sRNA corresponding length. Figure S2: Annotation of small RNAs derived from Solexa sequencing of silver carp small RNAs libraries. Figure S3: The number of differentially expressed known miRNAs. Table S1: Sequencing information on small RNAs and the distribution of sequences. Table S2: Information of known differently expressed miRNAs. Table S3: Significantly enriched biological processes for the candidate target genes of differentially expressed miRNAs by using GO enrichment analysis. Table S4: Enriched 20 KEGG pathways for the candidate target genes of differentially expressed miRNAs.
