# Supplementary material for: Aberrant Expressional Profiling of Known MicroRNAs in the Liver of Silver Carp (Hypophthalmichthys molitrix) Following Microcystin-LR Exposure Based on samllRNA Sequencing
Source: Toxins (Basel). 2020 Jan 9;12(1):41. doi: 10.3390/toxins12010041 (PMC7020426; doi:10.3390/toxins12010041)
Supplement: Supplementary file 1 [file toxins-12-00041-s001.zip › toxins-675101-supple-final/toxins-575101-supple-figures-final.docx]

Supplementary Materials: Aberrant Expressional Profiling of Known MicroRNAs in the liver of Silver Carp (*Hypophthalmichthys molitrix*) following Microcystin-LR Exposure Based on samllRNA Sequencing

Yiyi Feng, Xi Chen, Junguo Ma, Bangjun Zhang and Xiaoyu Li


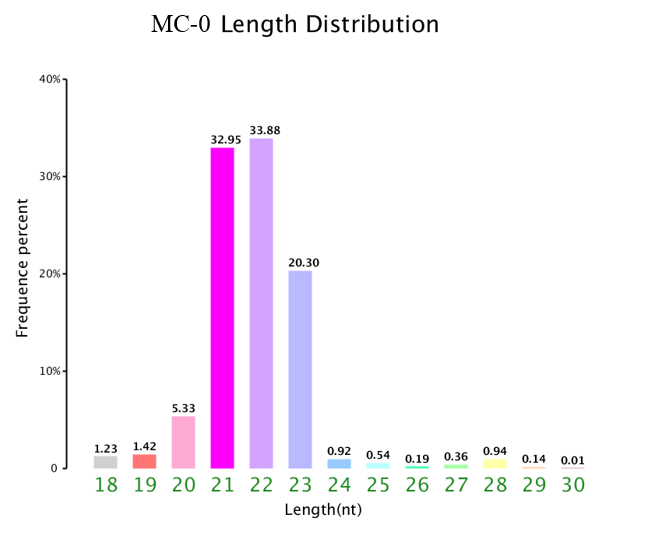

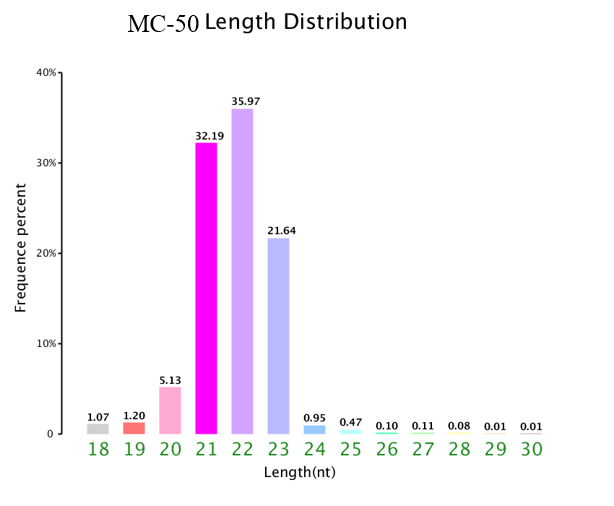


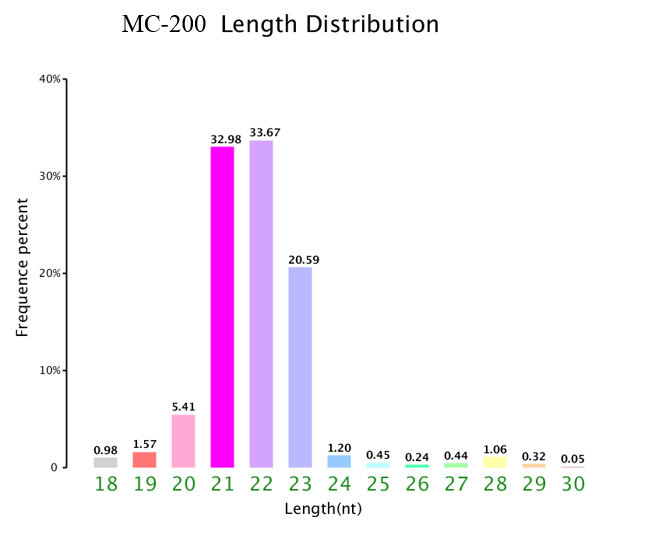


**Figure S1.** Small RNA length distribution. Abscissa: fragment length. Ordinate: proportion of sRNA corresponding length. Abscissa: segment length. Ordinate: proportion of sRNA of corresponding length


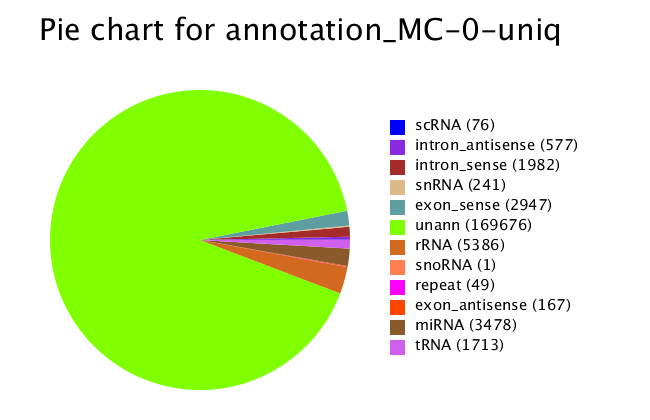

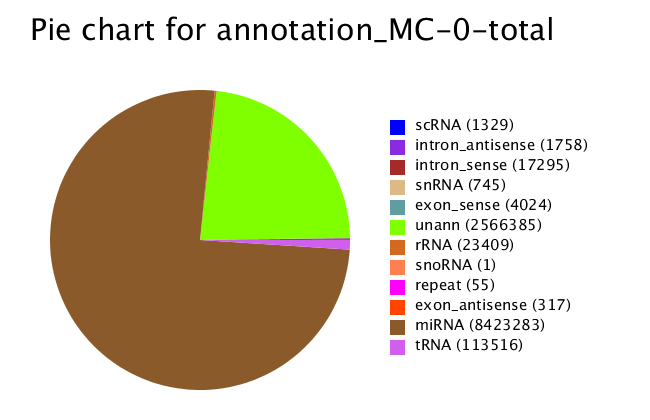


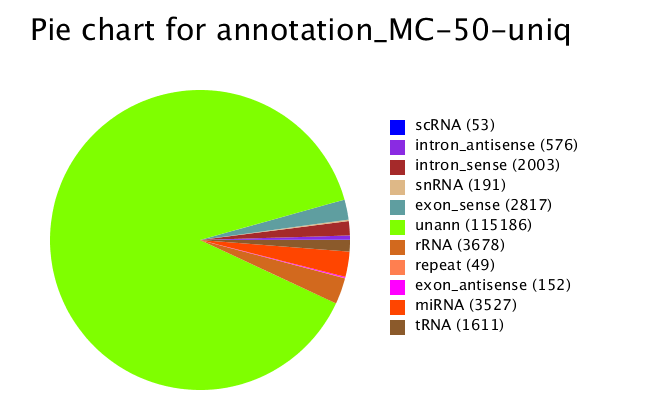

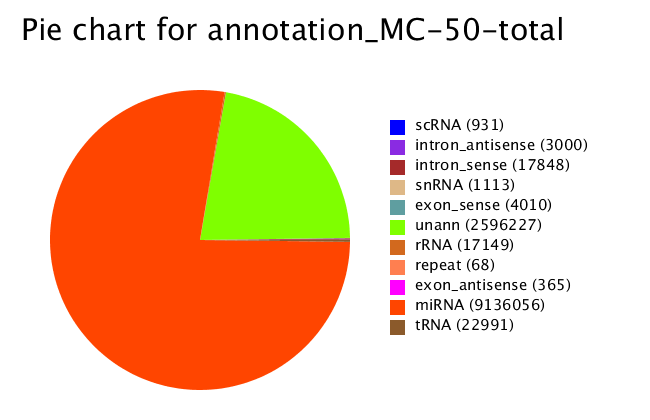


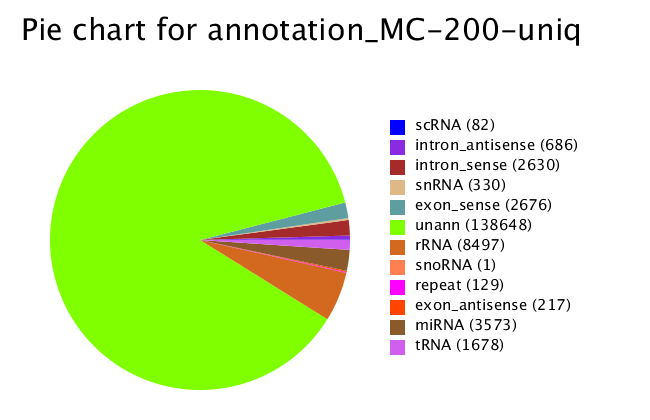

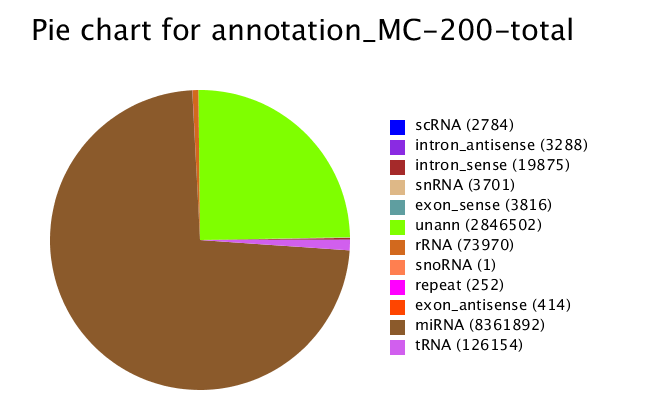


**Figure S2.** Annotation of small RNAs derived from Solexa sequencing of silver carp small RNAs libraries.

The comparison between all sRNA and various types of RNA was summarized: the left figure shows the species number of reads (uniq) compared to all kinds of non-coding sRNA; the right figure represents the total number of reads (total) compared to various types of non-coding sRNA


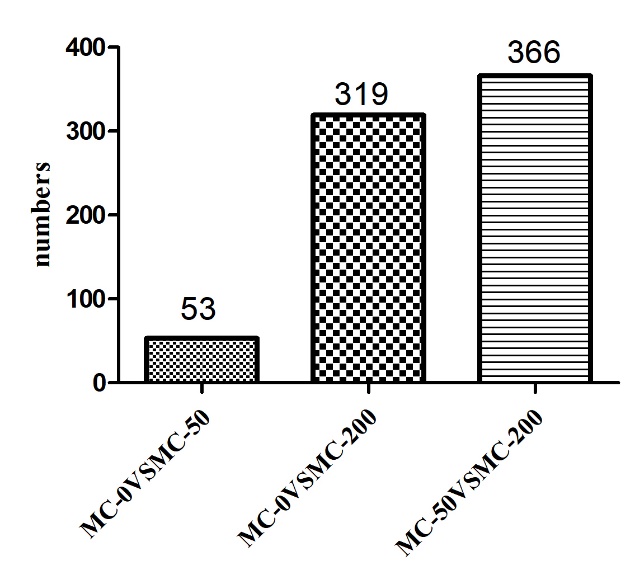


**Figure S3.** The number of differentially expressed known miRNAs. Abscissa: the number of known differently expressed miRNAs. Ordinate: comparison of concentration groups.
